# Biomaterials strategy for promoting palatal wound healing

**DOI:** 10.3389/fbioe.2025.1646629

**Published:** 2025-09-02

**Authors:** Wen Tong, Junxin Ren, Xinyi Yao, Siyi Wang, Wanhang Li, Liyang Zheng, Xiaoxia Hong, Shufan Zhao

**Affiliations:** ^1^ Institute of Stomatology, School and Hospital of Stomatology, Wenzhou Medical University, Wenzhou, Zhejiang, China; ^2^ Department of Oral Maxillofacial Surgery, School and Hospital of Stomatology, Wenzhou Medical University, Wenzhou, Zhejiang, China

**Keywords:** palatal wound healing, biomaterials, treatment, oxidative stress, inflammation

## Abstract

Palatal wounds arising from trauma, tumors, cleft palate, or free gingival grafting (FGG) and other etiologies compromise critical orofacial functions including mastication, deglutition phonation and articulation, while posing life-threatening risks in severe conditions. Although surgical resection remains the primary clinical intervention, current treatment strategies for palatal injuries are constrained by several limitations, including: bacterial contamination and chronic inflammation, extensive soft tissue defects, postoperative scar formation, compromised blood supply in the surgical field, and potential patient comorbidities. Research on wound healing based on biomaterials has advanced substantially in recent decades, significantly facilitating their application in tissue engineering. This review provides a comprehensive overview of biomaterials used in palatal wounds, including acellular dermal matrix (ADM), platelet-rich derivatives (e.g., PRF, PRP), amniotic membrane, growth factor, hyaluronic acid, collagen, novel hydrogel, nanofiber scaffolds and other relevant materials. It further discusses potential mechanisms that may be involved in palatal wound healing. The objectives of this review are to summarize recent advances in preclinical and clinical studies on biomaterials for palatal wound healing and to highlight their therapeutic potential in this context.

## 1 Introduction

Substantial variation exists in the incidence of palatal injuries across etiologies, peaking in surgical contexts such as cleft palate repair. Primary and secondary palatal fistulas are commonly associated with congenital anomalies such as cleft palate or as complications of surgical procedures. Reported rates of palatal fistula occurrence following cleft palate repair ranges from 5% to 30% (Eliason et al., 2020; Huang et al., 2023; Sakran et al., 2023; Tache and Mommaerts, 2019), with this variation primaly driven by factors such as surgical technique, patient adherence, and postoperative management. Such defects affect quality of life through impaired speech, swallowing, and oral hygiene. Given the palate’s intricate structure and functions, achieving physiological wound healing here poses unique clinical challenges. Achieving rapid and effective restoration of tissue integrity is crucial to restore speech and feeding functions and thereby mitigating long-term disability and socioeconomic burden, especially following extensive trauma or surgical intervention.

Current treatment options for palatal injuries are constrained by several limitations. Despite the efficacy in routine cases traditional surgical approaches, may lead to complications like wound dehiscence or fistula formation, particularly in patients with large clefts or compromised tissue quality. Existing biomaterials used in palatal repair also present inherent drawbacks. Some materials may exhibit inadequate sufficient mechanical strength or biocompatibility, resulting in suboptimal integration with surrounding tissues or eliciting immune responses. Additionally, the controlled release of therapeutic agents from these materials often requires complex fabrication processes and may not achieve the requisite spatiotemporal precision for optimal tissue regeneration.

In this review, literature was systematically searched in the PubMed database, covering publications from 2010 to the present. Both animal studies and clinical (human) studies were included to provide a comprehensive overview of the topic (Table 1).

**TABLE 1:** Summary of studies on biomaterials in palatal wound healing.

Major category	Biomaterial	Model	Site	Study Ref.
Acellular dermal matrix	ADM	Human	Hard palate	Simpson et al. (2019)
		Human		Matyskova et al. (2024)
		Yorkshire piglets		Kirschner et al. (2006)
Platelet-rich derivatives	PRF	Human	Hard palate	Femminella et al. (2016)
				Meza-Mauricio et al. (2021)
				Gusman et al. (2021)
				Patarapongsanti et al. (2019)
	PRP	Dog	Palate(double)	Shayesteh et al. (2012)
Amniotic membrane	AM	Human	Hard palate	Fénelon et al. (2018)
		Piglets		Rohleder et al. (2013)
		Berlin minipig	Mid-palate	Kesting et al. (2010)
Growth factor	Rh-EGF	Canine	Hard palate	Ben Amara et al. (2019)
	VEGF	Mice		Keswani et al. (2013)
	bFGF	Dog		Ayvazyan et al. (2011)
Hyaluronic acid	HA	Human	Hard palate	Yıldırım et al. (2018)
				Alpan and Cin. (2023)
				Joshi et al. (2024)
Collagen	Collagen	Human	Hard palate	Thoma et al. (2016)
Hydrogel	SSAD	Rat	Hard palate	Liu et al. (2022)
Nanofiber scaffolds	FTY720	Mice	Hard palate	Ballestas et al. (2019)
				Burnham et al. (2025)
Others	Proline-Rich Peptide(P2)	Rat	Hard palate	Villa et al. (2015)
	DOMG			Zhu et al. (2015)

In Summary, we aim to comprehensively analyze current biomaterials used in palatal wound healing, discuss the mechanisms of palatal wound healing, and assesses the therapeutic role of biomaterials in this regenerative process. By evaluating the advantages and limitations of different biomaterials while highlighting emerging tissue-engineering strategies, we aim to inform future research directions and advance clinical translation for improved management of palatal injuries, thereby optimizing functional outcomes and patient quality of life.

## 2 Biomaterials used in palatal wound healing

### 2.1 Acellular dermal matrix (ADM)

Acellular Dermal Matrix (ADM) is a decellularized natural biomaterial derived from skin tissues. Decellularization, achieved through physical, chemical, or biological techniques, removes cellular components (e.g., cellular debris, DNA.) while preserving the three-dimensional structure of the extracellular matrix (ECM) and its bioactive components (collagen, elastin, glycosaminoglycans) (Xing et al., 2015; Moore et al., 2015; Arellano et al., 2024). ADM finds broad application in burn wound repair, plastic surgery reconstruction, and oral mucosal defect repair (Chen et al., 2022; Zhao et al., 2023). It exhibits the advantages such as low immunogenicity, resistance to infection and reduction of scar formation, establishing it as a key material in soft tissue regeneration engineering (Zhao et al., 2023).

**FIGURE 1:**
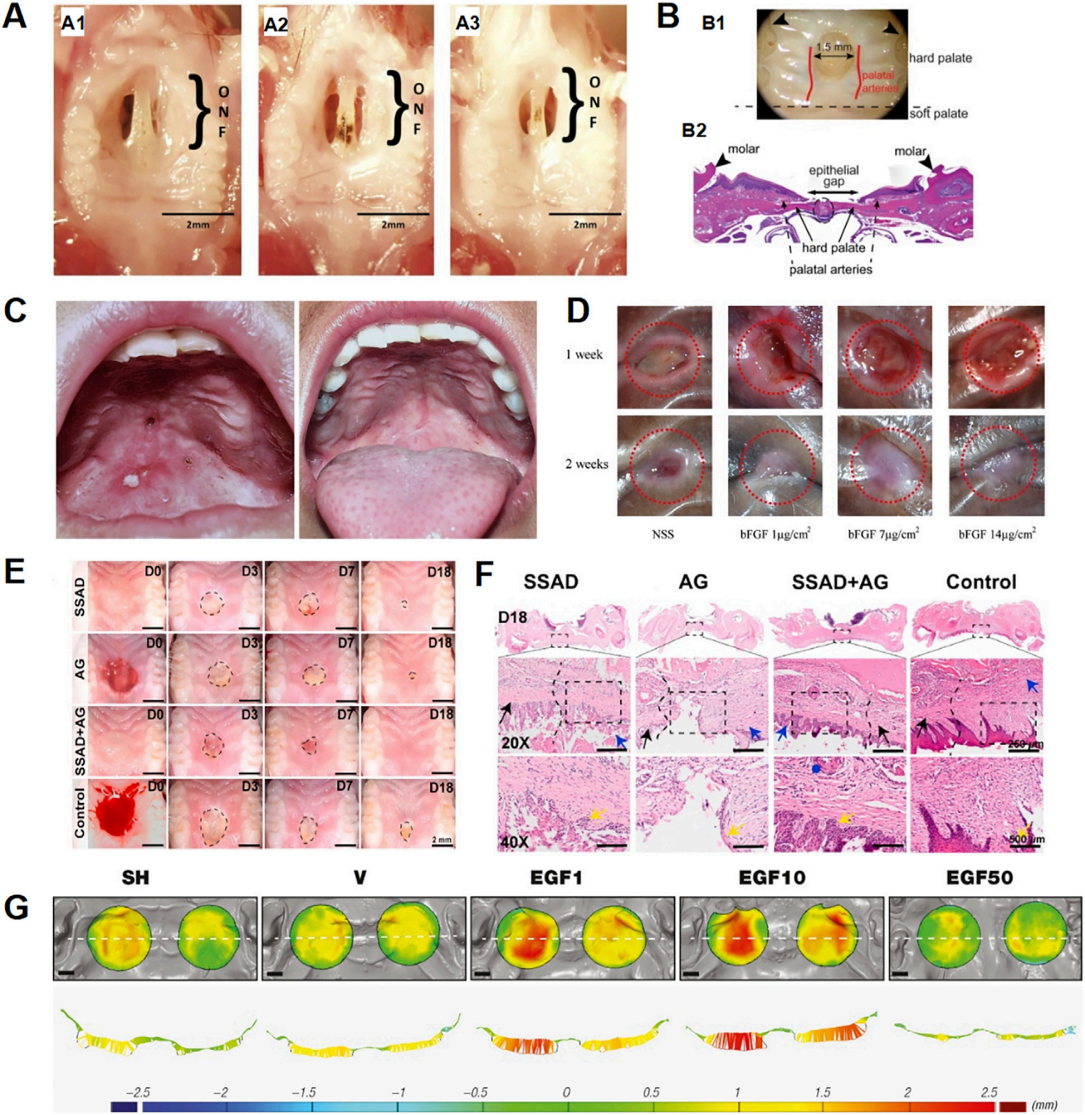
Studies on biomaterials in palatal wound healing. **(A)** Animal models for studying poor palatal wound healing and oronasal fistula: (A1–A3) Changes in palatal wound in mice created with cautery over 7 days, (A1) Palatal wound on day 3, (A2) Palatal wound on day 5, (A3) Palatal wound on day 7 (Ballestas et al., 2019); **(B)** Schematic of palatal wounding model and quantification of wound healing. (B1) Palate wounds were created in the hard palate. The wound site was standardized such that the anterior edge of the wound abutts an imaginary line drawn between the 1st molars (arrowheads) as anatomic reference. The size of the wound is 1.5 mm (B2) H&E-stained sections were analyzed for wound healing. Wounds with intact suture lines and minimal leukocyte infiltration were included. Epithelial gap was measured to assess closure. (Keswani et al., 2013); **(C)** (Left) Oronasal fistula before repair. (Right) Complete healing of oronasal fistula after repair with acellulardermal matrix.; **(D)** Macroscopic wound appearance at 1and 2 weeks post-implantation. All groups showed incomplete closure at 1 week. At 2 weeks, only the NSS group remained unepithelialized, while bFGF (7 and 14 mg/cm^2^) groups achieved complete closure; the 7 mg/cm^2^ group showed reduced contracture. Red circles: 6 mm diameter. (Ayvazyan et al., 2011); **(E,F)** Assessment of the wound healing rates in the palate. **(E)** Macroscopic observations of wounds. **(F)** New tissue thicknesses in different groups at 7 days postsurgery.; **(G)** Soft tissue volume changes from post-surgery to day16 in defects treated with vehicle (V), rhEGF at 1, 10, and 50 μg/g (EGF1, EGF10, EGF50), or spontaneous healing (SH). Occlusal views (top) and corresponding transverse sections (bottom) are shown. Color scale indicates thickness change: blue (decrease), green (no change), yellow/red (increase). Dashed line: transverse section reference; black bar = 2.5 mm. (Ben Amara et al., 2019).

The application of ADM in cleft palate repair garnered significant clinical interest. In 2018, Simpson A. et al. reported in a systematic review that the fistulation rate associated with ADM in primary cleft palate repair was 7.1%, while the recurrence rate following orofacial fistula closure was 11% (Simpson et al., 2019). In 2024, Matyskova D. et al. reported a case of secondary cleft palate repair using the MatriDerm^®^ ADM demonstrating significant improvement in wound healing and facilitating the patient’s transition from nasal to oral feeding (Matyskova et al., 2024). In 2006, Kirschner et al. (2006) demonstrated the efficacy of ADM in orofacial fistula repair utilizing both animal models and clinical cases, with all patients achieving successful healing following ADM repair (Figure 1C). Nevertheless, current evidence remains limited by the paucity of high-quality data supporting long-term outcomes and the suboptimal methodological rigor of existing studies (Simpson et al., 2019). Consequently, while ADM exhibits considerable potential for enhancing tissue healing and functional recovery, rigorously designed randomized controlled trials are warranted to definitively establish its clinical value.

### 2.2 Platelet-rich derivatives

The three-dimensional fibrin network of PRF, coupled with its sustained growth factor release, enhances cell proliferation, migration, angiogenesis, and wound healing (Ustaoğlu et al., 2016). In a clinical study assessing palatal donor site healing, Femminella B et al. treated 40 patients undergoing free gingival graft harvesting with platelet-rich fibrin and gelatin sponge wound dressings. Their findings demonstrated that PRF palatal bandages significantly accelerated wound healing and reduced postoperative pain (Femminella et al., 2016). In 2021, Meza-Mauricio, J. et al. reported in a systematic review that PRF membranes significantly accelerated early wound healing (≤2 weeks postoperatively), concurrently reducing postoperative pain and bleeding (Meza-Mauricio et al., 2021). Similarly, Gusman, D. J. et al. demonstrated that PRFs promote complete epithelialization of palatal wounds and significantly decreases postoperative pain (Gusman et al., 2021). In contrast, Shayesteh, Y.S. et al. observed no significant improvement in palatal wound healing with platelet-rich plasma (PRP) application (Shayesteh et al., 2012). Patarapongsanti, A.et al. compared PRF with oxidized regenerated cellulose (ORC) in palatal donor-area wound healing, and found that PRF was effective in reducing postoperative pain but finding no statistically significant difference in wound healing rates between the two materials (Patarapongsanti et al., 2019).

Clinical evidence supports platelet-rich fibrin (PRF) for palatal wound healing; however, significant methodological heterogeneity persists across studies regarding design, sample size, lesion characteristics, and outcomes. Investigations by Femminella et al., Meza-Mauricio et al., and Gusman et al. report accelerated epithelialization and reduced pain, but these conclusions derive from limited cohorts with variable follow-up and PRF protocols.

Current studies lack mechanistic validation, relying on subjective assessments (e.g., pain scales) without complementary biomarker or histological analyses. Patarapongsanti et al.‘s discordant findings—showing comparable healing between PRF and oxidized regenerated cellulose (ORC)—underscore the need for standardized, controlled trials. Platelet-rich plasma (PRP) shows reduced efficacy in palatal regeneration, likely due to rapid growth factor release and absence of a structured fibrin matrix. Direct PRF-PRP comparisons remain scarce, and preparation variations confound interpretation.

Collectively, the clinical translation of platelet-rich fibrin (PRF) is impeded by unstandardized preparation protocols, variability in biological composition, and the absence of unified evaluation metrics. Therefore, future clinical trials must prioritize protocol standardization, histologically validated endpoints, and adequate statistical power to conclusively demonstrate PRF’s therapeutic efficacy.

### 2.3 Amniotic membrane

Amniotic material demonstrates significant translational potential in the repair of orofacial fistula. Fénelon, M.et al. reported in a systematic review that amniotic membrane exhibits anti-inflammatory properties and good tissue integration for oral hard and soft tissue reconstruction (Fénelon et al., 2018). Building upon these mechanistic insights, Rohleder, N.H.et al. established the feasibility of multilayered amniotic membrane grafts for orofacial fistula repair using a porcine model and clinical cases. The results demonstrated a lower level of postoperative inflammation in the amniotic membrane transplantation group with complete healing in clinical cases (Rohleder et al., 2013). A study by Kesting, M.R.et al. further demonstrated that human amniotic membrane (HAM) grafts were significantly effective in repairing mid-palatal fistulas, with complete healing in two patients and a significant reduction in the diameter of the fistula in the other case (Kesting et al., 2010). These preliminary findings highlight three key limitations: restricted sample sizes across studies, heterogeneity in human amniotic membrane (HAM) preservation protocols, and the absence of standardized outcome metrics. To address these limitations, future studies should establish standardized preservation protocols incorporating quantitative endpoints (e.g., fistula area quantification, epithelialization rates) to facilitate cross-study comparability. Furthermore, multi-center randomized controlled trials featuring extended follow-up periods with adequate statistical power are warranted to enhance external validity.

### 2.4 Growth factor

Growth factors are secreted bioactive molecules that regulate cellular growth and proliferation. Key examples include platelet-derived growth factor (PDGF), epidermal growth factor (EGF), fibroblast growth factor (FGF) and erythropoietin (EPO). For this reason, Ben Amara et al. (2019) use human EGF (rh-EGF) to enhance the innate healing in oral mucosa wounds. The results demonstrated that rh-EGF accelerates early healing of soft tissue wounds in preclinical models by stimulating epithelial cell proliferation, inducing subepithelial neovascularization, and reducing inflammation (Figure 1G). However, this study was limited by its small sample size, lack of dose-response analysis, and requirement for clinical validation in humans. In complementary research, Keswani et al. (2013) suppressed salivary VEGF through submandibular gland excision (Figure 1B). Subsequent oral VEGF supplementation restored both angiogenesis and healing kinetics to baseline levels. While confirming VEGF’s critical role, the precise receptor-mediated mechanisms and synergistic interactions with other factors warrant further investigation. However, uncontrolled delivery raises safety concerns including risks of pathological angiogenesis and tumorigenesis, compounded by undefined receptor signaling mechanisms need to be further explored. In addition, Ayvazyan A et al. utilized bFGF-loaded collagen-gelatin scaffolds in standardized canine palatal wounds (Figure 1D), the researchers found that it significantly accelerated the wound contraction, epithelialization, and healing process by histological analysis (Ayvazyan et al., 2011). The specific molecular mechanism of bFGF in the healing process of palatal mucosal wounds needs to be further investigated. Future progress critically depends on three strategies: nanoparticle encapsulation to prolong growth factor half-life, stimuli-responsive hydrogels enabling spatiotemporally controlled release, and mechanistic elucidation of multi-factor synergistic networks.

### 2.5 Hyaluronic acid (HA)

Hyaluronic acid (HA), a high molecular weight, non-sulfated glycosaminoglycan ubiquitously present in body fluids such as synovial fluid, saliva, and gingival crevicular fluid, demonstrates therapeutic potential for palatal wound repair. A randomized controlled clinical trial (Yıldırım et al., 2018) evaluated the effect of HA on patient discomfort and wound healing in the palatal donor area after free gingival grafting by topical application. The results demonstrated that topical application of HA had a positive effect on postoperative pain and promoted wound healing in the palatal donor area. However, the study was limited to evaluating clinical parameters without histologic, biochemical, or immunologic analyses, and two different concentrations of HA were used in the study without exploring in detail the specific effects of the different concentrations on the healing process. Alpan and Cin. (2023) in a comparative study found HA significantly enhanced epithelial healing (measured by WHI/CE indices), outperforming hypochlorous acid (HOCL) and flurbiprofen—the latter two materials showed analgesic effects but potentially impaired tissue regeneration. Joshi, V.M. et al. demonstrated HA accelerated complete wound epithelialization, improved tissue color matching, and exhibited concentration-dependent efficacy (Joshi, V.M., et al., 2024). Persistent translational challenges include standardization deficits, as the optimal HA concentration has yet to be defined; delivery limitations, since topical formulations demonstrate poor mucosal retention with less than 40% remaining after 6 h; and mechanistic gaps, due to the largely uncharacterized molecular pathways involved in epithelial regeneration. Future research should focus on conducting dose-escalation trials to determine the therapeutic window, designing mucoadhesive delivery systems to extend mucosal residence time, and undertaking mechanistic investigations to link HA concentration with biomarkers of fibroblast migration and epithelialization.

### 2.6 Collagen

Collagen, the most abundant animal protein, exhibits excellent biocompatibility, biodegradability, and high tensile strength *in vitro*. These properties establish it as an ideal biomaterial for wound healing and tissue engineering. A randomized controlled clinical trial conducted by Thoma et al. (2016) collected quantitative tissue sections from symmetrical sites on the palate of volunteers and sutured a heterogeneous collagen matrix to one of the sites. Based on histological and immune-histological analyses, found that xenogeneic collagen matrix (XCM) was able to reduce granulation tissue formation and increase epithelial cell coverage compared to natural healing. This suggests that XCM have has a positive effect on reducing wound healing risks. However larger multi-center RCTs with standardized endpoints (e.g., re-epithelialization rate, scar quality) and mechanistic validation (*in vivo* cell migration assays) are essential to establish causal efficacy beyond this preliminary evidence.

### 2.7 Novel hydrogel

A study by Liu X et al. introduced a bioinspired adhesive hydrogel derived from the skin secretions of *Andrias davidianus* (SSAD) (Figures 1E,F), which demonstrated multifunctional regenerative properties, including: promotion of fibroblast and keratinocyte migration/proliferation, acceleration of extracellular matrix (ECM) deposition, recruitment of endogenous mesenchymal stem cells to wound sites, and sustained release of bioactive components—collectively enhancing wound healing. (Liu et al., 2022). In addition, the article applied SSAD hydrogel to a diabetic rat palatal mucosal defect and a cutaneous melanoma model, respectively, demonstrating its potential application in diabetes and melanoma treatment. However, this study is still deficient in drug release monitoring, cell specificity, and long-term effect assessment. Future studies should further optimize the experimental design and deeply explore its mechanism of action.

### 2.8 Nanofiber scaffolds

Nanofiber scaffolds represent a promising strategy for oral tissue regeneration, offering both structural support and controlled delivery of therapeutic agents. For instance, the use of FTY720-a sphingosine 1-phosphate receptor modulator, delivered from nanofiber scaffolds in the oral cavity has been shown to significantly enhance the healing of oronasal fistula (ONF). This local delivery strategy promotes complete wound closure, the researchers also hypothesized that it might increases the recruitment of pro-regenerative immune cells, such as Ly6Clo monocytes and M2 macrophages (Ballestas et al., 2019), which could accelerate the inflammatory response, tissue repair and angiogenic processes in wounds (Figure 1A). Burnham, A.J.et al. further demonstrated that phosphorylated FTY720 (FTY720P) significantly promoted the healing of oral mucosal defects by inducing macrophage polarization towards the M2 phenotype (Burnham et al., 2025). Although FTY720-based regenerative strategies demonstrate considerable potential, several limitations temper the interpretation and translational relevance of current findings. The mechanistic understanding remains incomplete, with key signaling pathways such as the S1P receptor–SOX2/Pax9/Pitx2 axis insufficiently characterized. Moreover, critical translational factors—including scalable GMP-compliant hydrogel production, material stability, and clinical applicability in vulnerable populations like pediatric patients—have not been adequately addressed. Immunological analyses are also limited, lacking advanced approaches such as single-cell sequencing to delineate FTY720’s impact on immune subsets like regulatory T cells. Addressing these gaps in future studies will be essential, with particular emphasis on developing stable and clinically viable delivery platforms, employing physiologically relevant models such as human organoids and large animals, elucidating the complex FTY720–immune–regeneration interplay, and thoroughly evaluating long-term safety alongside combinatorial therapeutic strategies.

### 2.9 Other materials

Oscar Villa et al. demonstrated that topical application of proline-rich peptide (P2) significantly accelerated re-epithelialization and neovascularization in palatal wounds (Villa et al., 2015). Tingting Zhu et al. revealed that dimethyl oxaloacetylglycine (DMOG) by stabilizing the expression of hypoxia-inducible factor-1α (HIF-1α), significantly accelerated palatal wound angiogenesis and healing of palatal wounds (Zhu et al., 2015).

Acellular dermal matrices (ADMs) and platelet-rich derivatives (e.g., platelet-rich fibrin and platelet-rich plasma) have established clinical utility, demonstrating favorable biocompatibility and relative ease of use. However, ADM adoption may be constrained by its significant cost. Amniotic membrane allografts and hyaluronic acid-based biomaterials represent additional clinically utilized options, offering well-recognized regenerative properties, albeit with variable physical characteristics that can impact handling and application. In contrast, advanced platforms such as hydrogels and nanofiber scaffolds, though currently predominantly confined to preclinical models, exhibit sophisticated functionalities including targeted immunomodulation and mitigation of oxidative stress. These emerging technologies possess considerable translational promise, yet necessitate rigorous validation through further preclinical and clinical studies. Collectively, this comparative landscape highlights a fundamental tension between the immediate clinical readiness of established materials and the advanced, innovative capabilities of emerging ones. This underscores the critical need for the development and application of robust, integrative evaluation frameworks to systematically guide optimal biomaterial selection for specific therapeutic goals.

In conclusion, biomaterials have demonstrated significant advantages in promoting palatal wound healing. These include optimizing the healing microenvironment by mimicking the extracellular matrix, enabling sustained release of bioactive molecules (e.g., growth factors and anti-inflammatory agents), and providing tunable mechanical properties with drug-loading capacity to support personalized treatment. Critically, the biocompatibility of both natural and synthetic materials helps reduce the risk of rejection. However, several limitations persist in this field. These include small sample sizes and a predominant reliance on animal models in existing clinical research, the absence of standardized evaluation criteria, and the fact that the underlying molecular mechanisms remain poorly understood. In addition, challenges in clinical translation—such as interspecies differences and unverified long-term safety—continue to hinder progress. All of these issues warrant further investigation in future studies.

In order to fully understand their therapeutic significance, it is necessary to explore in depth their mechanisms of interaction with the biological environment at the molecular and cellular levels. The following sections will detail how these materials regulate wound healing processes through mechanisms such as the regulation of oxidative stress, inflammation, and tissue remodeling.

## 3 Studies of the mechanism of biomaterial used in palatal wound healing

**FIGURE 2:**
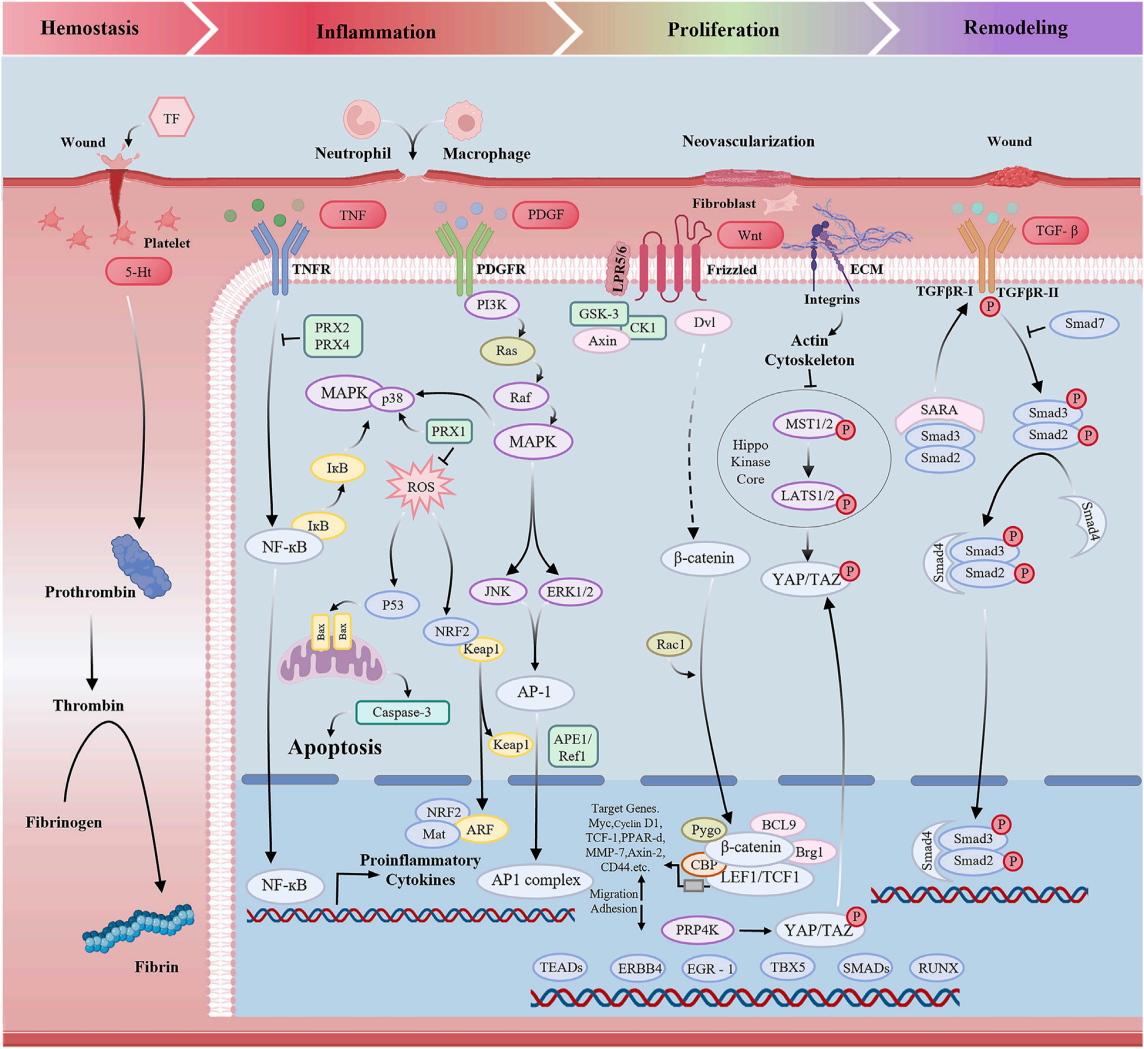
Wound healing-related signaling pathways.

Palatal wound healing is a complex and orderly biological process that includes multiple pathophysiological processes, including inflammatory, cell proliferation, cell migration, and tissue remodeling. However oxidative stress, excessive inflammation, impaired vascularization, and reduced collagen synthesis can significantly delay the healing process (Mohan et al., 2022). Therefore, a deeper understanding of the cellular and molecular mechanisms that orchestrate palatal wound repair is critical (Figure 2).

### 3.1 Oxidative stress

Oxidative stress refers to the excessive accumulation of reactive molecules, such as reactive oxygen stress (ROS) and reactive nitrogen stress (RNS) which occurs during the clearance of senescent cells or in response to various harmful stimuli, including injury, ultraviolet radiation, and toxic agents. When the production of oxidants exceeds the antioxidant defense capacity, a redox imbalance occurs, ultimately leading to oxidative damage to cellular structures and tissues. Wound healing involves four stages: hemostasis, inflammation, proliferation and remodeling (Guo and Dipietro, 2010; Ridiandries et al., 2018; Rodrigues et al., 2019). Recent studies have demonstrated that scavenging excessive ROS from the wound microenvironment can facilitate tissue regeneration and accelerate the healing process (Kang et al., 2024; Qu et al., 2024; Wang et al., 2024a; Wang et al., 2024b). Although direct evidence on ROS signaling in palatal wound healing is scarce, we hypothesize based on general wound models that oxidative stress also plays a critical regulatory role in this context. Although ROS are often regarded as detrimental molecules, low to moderate levels play beneficial roles in various physiological processes, including pathogen clearance, wound healing, and tissue repair. Moreover, ROS serve as essential signaling molecules in numerous cellular pathways (Bhattacharyya et al., 2014).

It was found that ROS-activated signaling pathways include p53-mediated apoptosis (Niwa-Kawakita et al., 2017), the mitogen-activated protein kinase (MAPK) pathway, nuclear factor erythroid 2-related factor 2 (NRF2)-mediated antioxidant response element (ARE) regulation, and nuclear factor-κB (NF-κB) activation (Morgan and Liu, 2011). Kozyrska et al. (2022) highlighted the pivotal role of epithelial cell repair, showing that after injury, epithelial cells undergo collective migration directed by specialized leader cells. Recent studies identified p53 as a key regulator of this process, promoting migration during repair and subsequently mediating the elimination of leader cells via cellular competition to restore epithelial homeostasis. These findings reveal a novel role for p53 in epithelial regeneration and suggest potential therapeutic targets for wound healing. Furthermore, angiogenesis plays a vital role in wound healing by regenerating blood vessels to ensure adequate oxygen delivery for tissue repair. Yuan et al. (2018) demonstrated that cinnamaldehyde promotes HUVEC proliferation, migration, and tube formation, thereby enhancing angiogenesis and upregulating VEGF secretion. This effect is mediated via activation of the PI3K/AKT and MAPK pathways, which regulate multiple stages of angiogenesis. Furthermore, biomaterial-based strategies have demonstrated the capacity to modulate redox status and associated signaling. Yang et al. (2023) developed a microneedle system based on hyaluronic acid embedded with ZCO nanomaterials for diabetic wound treatment. Although these findings were not obtained in palatal tissues, they support the rationale for developing antioxidant-releasing biomaterials, such as hydrogels or microneedles, as promising interventions for controlling oxidative stress and improving healing outcomes in palatal wounds. Future research should focus on validating these mechanisms in site-specific models.

### 3.2 Excessive inflammation and immunity

Another common factor that affects palatal wound healing is excessive inflammation. Numerous studies have demonstrated that chronic wounds exhibit an excessive inflammatory response (Huang et al., 2022; Dong et al., 2023; Ye et al., 2023; Zhang et al., 2023). Chronic wounds exhibit dysregulated inflammation, characterized by elevated levels of inflammatory cells (e.g., neutrophils, macrophages), proinflammatory cytokines, proteases (MMPs, elastase), and reactive oxygen species (ROS), alongside reduced protease inhibitors (e.g., TIMPs) (Ellis et al., 2018). Macrophages dominate the inflammatory phase of oral healing (Koh and DiPietro, 2011). M1 macrophages secrete pro-inflammatory cytokines and chemokines, including interleukin-6 (IL-6), interleukin-12 (IL-12), interleukin-1β(IL-1β) and tumor necrosis factor-α (TNF-α), which enhance the immune response to eliminate pathogens (Politis et al., 2016). M2 macrophages primarily secrete anti-inflammatory cytokines such as arginase-1(Arg1), interleukin-10 (IL-10) (Biswas and Mantovani, 2010) and TGF-β, which help suppress inflammation and play a crucial role in wound healing and tissue repair. Therefore, an excessive inflammatory response may lead to chronic wound formation and delayed healing.

Besides, there is a strong link between inflammation and immunity (Xu and Larbi, 2018). The immune system protects the body by identifying and eliminating pathogens, while inflammation is its immediate response to injury or infection. Upon activation, leukocytes migrate to affected sites, where they remove pathogens and damaged tissue through phagocytosis and release inflammatory mediators such as cytokines and chemokines. These mediators induce vasodilation and vascular permeability, leading to redness, swelling, heat, and pain. They also promote immune cell recruitment to infection sites. The adaptive immune system establishes memory via clonal expansion of antigen-specific lymphocytes, enabling rapid secondary responses. Additionally, immune cells secrete anti-inflammatory factors (e.g., IL-10, TGF-β) to resolve inflammation and maintain homeostasis. Thus, the immune system precisely regulates inflammation to eliminate threats and initiate tissue repair.

### 3.3 Regeneration

The transforming growth factor-β (TGF-β) signaling pathway orchestrates pivotal and diverse functions in wound healing and tissue regeneration by regulating essential cellular repair processes. Upon injury, TGF-β released from platelets and tissue-resident cells binds to cell surface type I and type II receptors, triggering the phosphorylation of SMAD proteins, particularly SMAD2 and SMAD3. The phosphorylated SMADs complex with SMAD4, translocate to the nucleus, and activate transcription of target genes regulating cell proliferation, differentiation, and extracellular matrix (ECM) synthesis (Chen et al., 2024). This cascade governs key cellular events such as fibroblast-to-myofibroblast differentiation—critical for collagen deposition and wound contraction. Furthermore, TGF-β modulates ECM remodeling by stimulating MMPs and inhibiting TIMPs, thereby balancing deposition and degradation essential for tissue repair.

In addition to its central role in wound healing, TGF-β signaling influences the inflammatory phase of tissue regeneration. It modulates the inflammatory response by modulating the activation and recruitment of immune cells such as macrophages, which subsequently secrete additional growth factors and cytokines that promote both the resolution of inflammation and the progression to tissue regeneration. The profibrotic capacity of TGF-β becomes particularly relevant in chronic wounds, where excessive signaling may result in pathological fibrosis, as observed in hypertrophic scars and keloids (Li et al., 2025).

In addition to TGF-β, other signaling pathways critically contribute to tissue regeneration. The Wnt/β-catenin pathway, for instance, regulates stem cell lineage commitment and self-renewal—particularly in epithelial and mesenchymal tissues. Through β-catenin stabilization, this pathway activates transcription of regenerative genes, thus driving cellular proliferation and differentiation essential for tissue repair (Yu et al., 2024). Similarly, the Notch signaling pathway is instrumental governs cell fate decisions, particularly during endothelial cell differentiation and angiogenesis, key processes for vascularized tissue regeneration. Notch activation occurs via ligand-receptor binding, triggering proteolytic cleavage that releases the Notch intracellular domain (NICD) to modulate differentiation-related gene expression (Zhou et al., 2022).

The Hippo-YAP/TAZ pathway serves as a pivotal regulator of tissue homeostasis and regeneration. This pathway governs cell proliferation, survival, and organ size by modulating the activity of the YAP/TAZ transcriptional co-activators in response to mechanical signals and cell density (He et al., 2020). By integrating environmental signals, Hippo signaling modulates tissue regeneration—particularly in the liver, skin, and heart—by balancing cellular growth and apoptosis.

Finally, the fibroblast Growth Factor (FGF) signaling pathway plays a crucial role in promoting angiogenesis, fibroblast proliferation, and ECM synthesis, all of which are fundamental to tissue regeneration. Upon binding to its receptors, FGF activates downstream signaling cascades—most notably the MAPK and PI3K-Akt pathways—that enhance cell survival, migration, and proliferation in response to injury (Upadhayay, S., et al., 2025).

In summary, tissue regeneration is a highly coordinated process involving multiple signaling networks, including TGF-β, Wnt/β-catenin, Notch, Hippo-YAP/TAZ, and FGF pathways, each of which regulates key aspects of cellular behavior necessary for effective tissue repair and remodeling. Understanding the interplay between these pathways offers significant potential for therapeutic strategies aimed at enhancing tissue regeneration.

## 4 Potential biomaterials for palatal wound healing

The field of biomaterials is advancing rapidly, with applications spanning tissue engineering, regenerative medicine, and precision drug delivery. In the context of palatal wound healing, current research has progressed beyond structural support alone, focusing increasingly on functional biomaterials that actively modulate the wound microenvironment. These materials are designed not only to provide mechanical stability, but also to orchestrate key biological processes, such as oxidative stress reduction and angiogenesis. Emerging platforms including immunomodulatory hydrogels, bioactive nanofiber scaffolds, and stimuli-responsive delivery systems hold promise for enhancing tissue regeneration in the oral cavity.

### 4.1 Scaffold design for tissue engineering

Researchers are developing biodegradable and biocompatible scaffolds that can support cell growth, differentiation, and tissue regeneration (Gupta et al., 2014; Zhu et al., 2019). These scaffolds are designed to mimic the extracellular matrix (ECM) and provide a three-dimensional environment for cells. Biological scaffold design is an evolving field and current research is focused on meeting clinical needs by improving the biosafety and functionality of these materials.

### 4.2 Drug delivery systems

**FIGURE 3:**
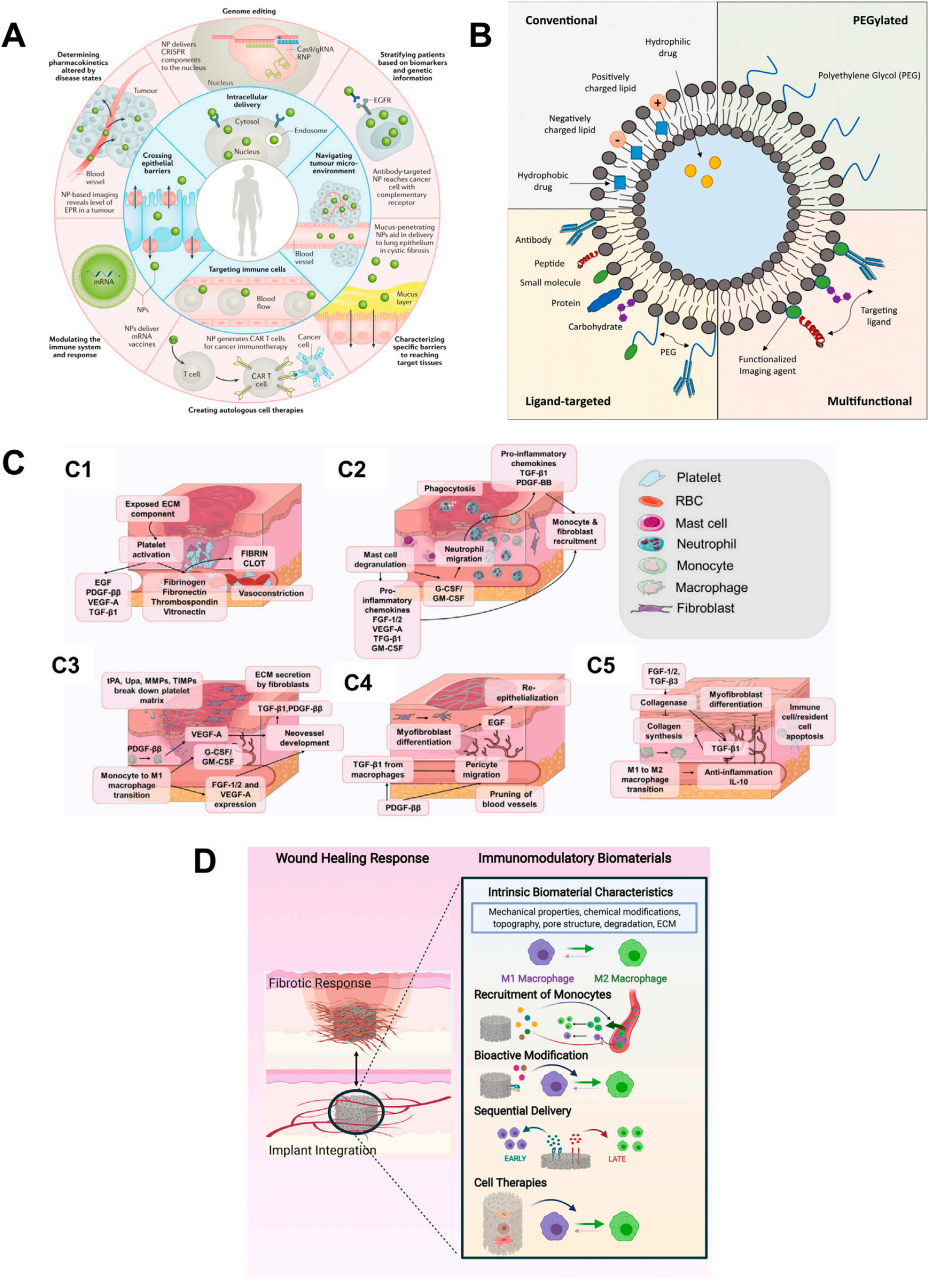
Current studies on biomaterials. **(A)** Overview highlighting some of the biological barriers that nanoparticles (NPs) can overcome (inner ring) and precision medicine applications that may benefit from NPs (outer ring). Intelligent NP designs that improve delivery have the potential to enhance the performance of precision medicines and, thus, accelerate their clinical translation. (Mitchell et al., 2021); **(B)** Different types of liposomes used in therapeutic applications. (Guimarães et al., 2021); **(C)** The process of wound healing: (C1) Hemostasis (C2) Inflammation (C3) Granulation Tissue Formation. (C4) Wound Contraction and re-epithelialization. (C5) Remolding (Mo et al., 2023); **(D)** Design strategies for immunomodulatory biomaterials that achieve implant integration. Most strategies focus on manipulation of biomaterial-intrinsic properties, the addition of bioactive factors that mediate recruitment, and/or the M1-to-M2 transition of macrophages, or the inclusion of immunomodulatory cell types (Whitaker et al., 2021).

Biomaterials are being engineered for controlled and targeted drug delivery. These includes nanoparticles (Figure 3A) (Mitchell et al., 2021) liposomes (Figure 3B) (Guimarães et al., 2021), hydrogels (Sanjanwala et al., 2022), and microneedle patches (Mo et al., 2023) that enable sustained and localized release of therapeutic agents. Mitchell et al. (2021) reviewed the substantial potential of nanoparticles in enhancing disease diagnosis and therapeutic precision, highlighting their ability to overcome the limitations of conventional drug delivery systems, such as poor biodistribution and inefficient intracellular transport. And they also discussed the application of nanoparticles in precision medicine to achieve customized design for precise applications in response to patient heterogeneity. Liposomes enhance the therapeutic efficiency and reduce systemic toxicity by stabilizing compounds, enhancing cellular and tissue uptake, and improving drug biodistribution throughout the body (Guimarães et al., 2021). Sanjanwala et al. (2022) described the advantages of polysaccharide-based hydrogels (PBHs) over synthetic hydrogels, such as non-toxicity, high biocompatibility and *in vivo* degradability. The application of PBHs in wound healing, especially chitosan-based wound dressings, which have wound healing-promoting and antimicrobial properties, was also discussed. However, the article points out that despite the many potential applications of PBHs in drug delivery and wound healing, translation from the laboratory to clinical applications remains a challenge. In a review on the role of microneedle (MN) array patches as novel wound dressings in modulating the wound microenvironment, Mo et al. (2023) mentioned that microneedle array patches, as a new type of wound dressing, enable non-invasive percutaneous drug delivery and can be combined with smart materials to enable real-time monitoring of the wound site and responsive release of therapeutic molecules (Figure 3C). Recent advances in smart biomaterials have moved beyond conventional delivery strategies toward platforms with enhanced functionalities. Li, J., and Kataoka, K. developed DNA-based nanorobots that respond to molecular cues in the tumor microenvironment, enabling highly specific drug release and reducing off-target effects (Li and Kataoka, 2021). Moreover, next-generation hydrogels have been designed to exhibit responsiveness to physiological stimuli such as pH, temperature, enzymes, or reactive oxygen species (ROS), thereby achieving spatiotemporal control over drug delivery. Wen P., et al. reviewed multifunctional hydrogel systems capable of dynamically interacting with the wound microenvironment by scavenging ROS, generating oxygen, and enabling responsive drug release (Wen et al., 2023). These innovations represent a shift toward programmable and adaptive drug delivery systems that can be tailored to patient-specific conditions, advancing the field of personalized medicine.

### 4.3 Bioactive coatings

The development of bioactive coatings for medical devices, such as implants and prosthetics, aims to enhance tissue integration and reduce the risks of infection and rejection. Oliver et al. (2019) reported that bioactive coated glass materials exhibit superior bioactivity and biological functions, including promotion of tissue regeneration and degradation rates similar to those of tissue regeneration, compared to traditional metallic biomaterials. The materials have shown to improved performance over conventional hard-tissue bonding in dental and orthopedic implants and demonstrate potential in soft-tissue applications. Furthermore, by varying the composition of the bioactive glass, additional functionalities can be introduced, such as enhanced osteogenesis through the addition of Sr2+, promotion of angiogenesis by Cu2+, and antimicrobial properties achieved by Ag+.

### 4.4 Immunomodulatory biomaterials

Designing biomaterials that modulate the immune response is crucial for applications in transplantation and chronic wound healing. These materials can be tailored to reduce inflammation and promote tissue integration. The study by Whitaker R et al. (Whitaker et al., 2021) reported biomaterials promote tissue repair by regulating immune cell behavior—particularly the activation of macrophages and monocytes and their interactions with other wound-related cells (Figure 3D). By controlling the structure of biomaterials and the release of immunomodulators, macrophage polarization from the pro-inflammatory M1 to the anti-inflammatory M2 phenotype can be facilitated, thereby accelerating healing. However, challenges remain in clinical translation, particularly the limitations of *in vitro* and animal models in replicating human immune responses.

### 4.5 Environmental and stimuli-responsive biomaterials

Stimuli-responsive materials react to environmental cues such as pH, temperature, or light, enabling applications in smart drug delivery and self-healing systems. Peng et al., (2023) developed a pH- and temperature-responsive microgel-embedded hydrogel (microgel@PAM/CS) that enables controlled drug release and wound healing. As a multifunctional material, microgel@PAM/CS holds promise for biomedical engineering, particularly smart wound dressings. However, its long-term safety and efficacy remain uncertain due to the lack of *in vivo* validation.

The oral cavity presents a uniquely dynamic and challenging environment for biomaterial performance, characterized by constant exposure to saliva, enzymatic activity, and a diverse microbial community. To ensure effective immunomodulation in such conditions, biomaterials must be adapted accordingly. Potential strategies include surface functionalization with anti-biofouling or antimicrobial moieties (e.g., zwitterionic groups, silver nanoparticles) to limit microbial colonization and preserve immune modulatory cues. Materials with enzyme-resistant backbones or saliva-tolerant degradation profiles may prolong *in situ* bioactivity. Furthermore, incorporating bioactive signals such as IL-4 mimetics, resolvins D1, or ECM-mimetic ligands can help maintain M2-polarizing activity even in the presence of oral microbiota. Future development of such oral-adapted immuno-instructive biomaterials will be crucial to translating macrophage-targeted strategies to clinical applications in palatal and other oral wound sites.

Notably, personalized biomaterial approaches are emerging as promising paradigms to enhance precision in oral tissue regeneration. For palatal repair, 3D-bioprinted scaffolds enable patient-specific anatomical precision, enhanced tissue integration, and spatiotemporally controlled therapeutic delivery. Individualized dosing of growth factors—informed by wound severity, patient age, or comorbidities like diabetes—may further optimize outcomes while mitigating off-target effects. Critically, although these approaches remain predominantly preclinical, their integration into scaffold design frameworks holds significant potential to address inter-patient heterogeneity and advance precision-guided oral regenerative therapies.

## 5 Summary

Despite significant advancements, several challenges hinder the clinical translation of biomaterial innovations. There remains a need for biomaterials that better replicate the complex environment, accounting for factors such as saliva, fluctuating pH, and mechanical forces. Moreover, the customization of biomaterials to individual patient needs, including personalized drug delivery and growth factor release, is an area requiring further exploration. In conclusion, biomaterials strategy for promoting palatal wound healing represents a promising avenue for the future. In this article, we present research on biomaterials in palatal wounds and point out the prospects for promoting palatal wound healing. Future research will focus on in-depth study of the molecular mechanisms of palatal wound healing and the design of biomaterials that promote wound healing by targeting specific mechanisms.
